# Homologous recombination repair gene mutations as a predictive biomarker for immunotherapy in patients with advanced melanoma

**DOI:** 10.3389/fimmu.2022.871756

**Published:** 2022-08-03

**Authors:** Zhixuan You, Meng Lv, Xuanyu He, Yingqin Pan, Junfeng Ge, Xue Hu, Yating Zheng, Mengli Huang, Chengzhi Zhou, Changxuan You

**Affiliations:** ^1^ Guangzhou Medical University, Guangzhou, China; ^2^ Department of Oncology, Huiqiao Medical Center, Nanfang Hospital, Southern Medical University, Guangzhou, China; ^3^ Department of Oncology, Nanfang Hospital, Southern Medical University, Guangzhou, China; ^4^ Medical Department, 3D Medicines Inc., Shanghai, China; ^5^ State Key Laboratory of Respiratory Disease, National Clinical Research Centre for Respiratory Disease, Guangzhou Institute of Respiratory Health, First Affiliated Hospital, Guangzhou Medical University, Guangzhou, China

**Keywords:** HRR, anti-CTLA-4 therapy, TMB, DDR, immune microenvironment

## Abstract

**Background:**

Nowadays, immunotherapy targeting immune checkpoint receptors is one of the cornerstones of systemic treatment in melanoma. Homologous recombination repair (HRR) is one of the DNA damage response (DDR) pathways, which has been proved to correlate with the efficacy of platinum-based chemotherapy, PARP inhibitor therapy, and immunotherapy in a variety of cancers. However, their predictive value of HRR remained unknown in patients with advanced melanoma.

**Methods:**

Data of advanced melanoma patients from an independent cohort (Samstein2018) were used to analyze the correlation with immunogenic markers and the prognostic effect of HRR on immunotherapy, and another four cohorts (pooled cohort: Miao2018, Allen 2015, Hugo2016, and Synder2014) were used for validation. Immune infiltration cell scores analyzed by TCGA-SKCM cohort were used to explore potential mechanisms related to the immune microenvironment.

**Results:**

Compared to patients with an HRR wild type (HRRwt), those with HRR mutations (HRRmut) in anti-CTLA-4-treated patients of the Samstein2018 cohort had higher tumor mutation burden (TMB; P = 0.0041) and longer median overall survival (mOS; P = 0.0094). In terms of results validation, it was also confirmed that the mOS (P = 0.0014) of HRRmut patients receiving anti-CTLA-4 therapy was significantly better than that of HRRwt patients in the pooled cohort, and objective response rates (ORR; P = 0.0053) were also found to be significant. However, there was no significant difference in mOS between HRRmut patients who received anti-PD-1/L1 therapy and HRRwt patients in either the discovery (Samstein2018 cohort, P = 0.94) or validation (pooled cohort, P = 0.96) set. Exploratory analysis found that although HRRmut patients showed no significant difference in mOS between anti-CTLA-4 and anti-PD-1/L1 therapy (P = 0.79), the mOS value of the anti-CTLA-4 therapy group (31.7 months) in HRRmut patients was numerically superior to the anti-PD-1/L1 therapy group (27.5 months). In contrast, the mOS of the anti-CTLA-4 therapy group was significantly lower than that of the anti-PD-1/L1 therapy group (12.4 vs. 32.0 months) in HRRwt patients. In addition, transcriptome profiling analysis revealed that the 29 (65.9%)-gene mutation of the HRR pathway associated with reshaping of the immunological microenvironment in melanoma.

**Conclusions:**

HRR mutations were associated with a higher TMB level, and better anti-CTLA-4 therapy outcomes. HRR may serve as an independent predictor of anti-CTLA-4 therapy efficacy in patients with advanced melanoma and their clinical value warrants further investigation.

## Introduction

The emergence of immune checkpoint inhibitors (ICIs) has changed the therapeutic paradigm of melanoma (1). Targeting either PD-1/PD-L1 or CTLA-4, the ICIs can improve overall survival (OS) and progression-free survival (PFS) and increase the long-term survival rate in patients with advanced melanoma (2–4). Therefore, ICIs are currently the standard of care in the systemic treatment of advanced melanoma (5).

Although ICIs significantly increased the survival of advanced melanoma, there are still more than 40% of patients who have no significant response and there is a considerable proportion of responder experience tumor relapse within 2 years (6). Therefore, identifying biomarkers to select patients who are more likely to benefit from ICIs is vitally important. Several genomic characteristics including high neoantigen load, high mutational load, and tumor clonality have been revealed to be predictive of a favorable response to anti-CTLA-4 therapy in melanoma (7, 8). As for anti-PD-1 blockade, high PD-L1 expression and tumor mutation burden have been recognized as predictors in melanoma and other solid tumors (9).

As a collective term, DNA damage response (DDR) refers to the induction and detection of DNA damage that affects a plethora of intracellular and intercellular signal transduction events and enzyme activity (10). Previous studies have shown that the DDR pathway is associated with chemotherapy, targeting, and immunotherapy for multiple cancer types (11–13). For immunotherapy, DDR deficiency has been an important determinant of tumor immunogenicity in recent studies, which may promote antigenicity through increased mutability and genomic instability (14). The DDR system comprises eight pathways, namely, mismatch repair (MMR) base excision repair (BER), checkpoint factors, Fanconi anemia (FA), homologous recombination repair (HRR), nucleotide excision repair (NER), non-homologous end-joining (NEJ), and DNA translesion synthesis (TLS) (15). Existing research shows that the role of each pathway in the immune system is different. For example, mutations in the genes of the MMR pathway can predict the immunotherapy benefit of patients with colorectal cancer (16) and HRR defects may be a potential predictive biomarker of response to the PD-1 inhibitor in metastatic castration-resistant prostate cancer (17).

The HRR pathway is one of the most important parts of DDR, which has a guiding significance in the treatment of solid tumor (18). The exploration of the relationship between HRR gene and the therapies of solid tumors originated from the emergence of PARP inhibitors, and the HRR correlation with the efficacy of PARP inhibitors has also been reported in melanoma (19). In addition, gene mutations in the HRR pathway have been reported associated with immunotherapy efficacy in pancreatic, gastric, colon, breast, ovarian, and lung cancers, but none has been reported in melanoma (20–25). Therefore, it is significant to analyze the relationship between HRR pathway and immunotherapy in melanoma. In this study, we used five public cohorts of melanoma to explore the association between HRR pathway gene mutations and response to immunotherapy, including anti-CLTA-4 and anti-PD-1/L1 therapy.

## Materials and methods

### Clinical cohorts and study design

We searched literatures and found five advanced melanoma cohorts (**Table 1**) treated with anti-CTLA4/anti-PD-(L)1, which were Samstein2018 (PMID: 30643254), Miao2018 (PMID: 29301960), Allen 2015 (PMID: 26359337), Hugo2016 (PMID: 26997480), and Synder2014 (PMID: 25409260), with a total of 452 advanced melanoma patients with gene sequencing and clinical data. We downloaded whole exon sequencing (WES), gene expression data, and clinicopathologic information of the Miao cohort, Allen cohort, Hugo cohort, Synder cohort, and targeted-sequencing data and overall survival data of the Samstein cohort from the cBioPortal database (https://www.cbioportal.org/ ). Data of the Miao cohort were obtained from the previous published research (26).

**Table 1:** Statistical results of the number of patients in four cohorts that make up the pooled cohort.

Cohort	No. of Pts	No. of Mut
Miao2018.Pancancer.249.WES	147	75
Melanoma.Allen2015.WES.110	110	37
Melanoma.Hugo2016.WES.38	37	17
Melanoma.Synder2014.WES.64	61	31
Total	355	160

**Figure 1:**
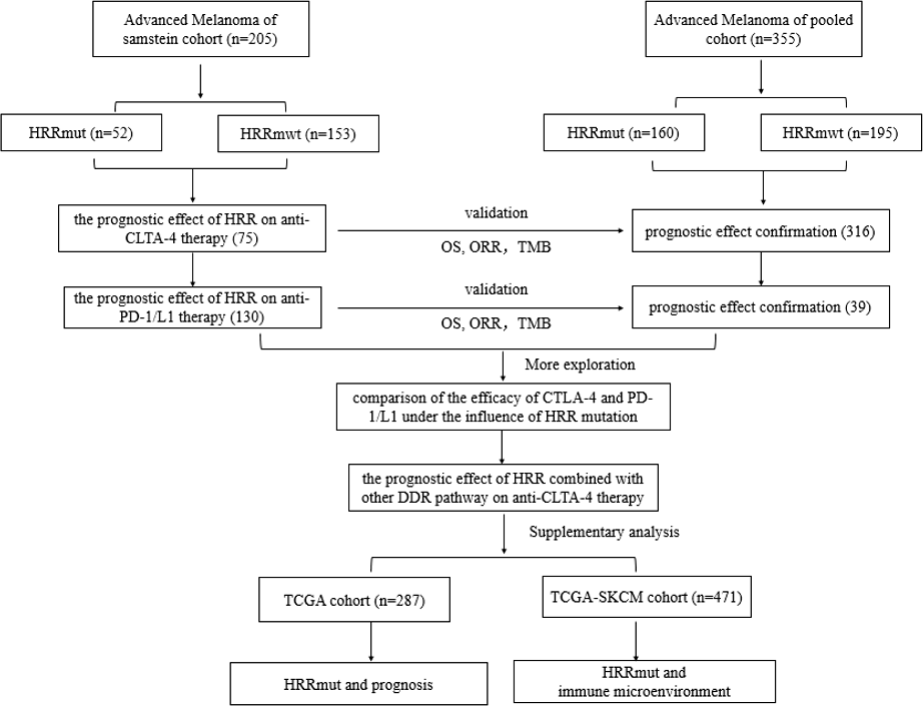
Research design roadmap.

As shown in **Figure 1**, data of 205 patients with advanced melanoma from an independent cohort (Samstein2018) (27) were used to analyze the correlation with immunogenic markers and the prognostic effect of HRR on immunotherapy. Data of 355 patients of another four cohorts (pooled cohort: Miao2018, Allen 2015, Hugo2016, and Synder2014) (7, 8, 26, 28) were used for the results validation. In order to distinguish between HRR mutations’ prognostic role, data from 287 untreated advanced melanoma patients in The Cancer Genome Atlas (TCGA) database were analyzed. TCGA dataset was accessed *via* cBioPortal (www.cbioportal.org/). In addition, the fragments per kilobase million mapped reads (FPKM) from TCGA-SKCM cohort (including 471 advanced melanoma patients) were transformed into transcripts per kilobase million (TPM) values. Estimation of immune infiltration cell scores was conducted *via* TIMER2.0 (http://timer.cistrome.org/) using TPM data.

Forty-four genes were identified as HRR pathway genes (**Supplementary Table 1**) and other DDR pathway genes (**Supplementary Table 2**) based on searches of the PubMed, NCBI Gene, and Biosystems databases.

### Determination of deleterious HRR and other DDR mutation status

Non-synonymous mutations including TRUNC (frameshift del, frameshift ins, nonsense, nonstop, splice region, and splice site), INFRAME (inframe del and inframe ins), and MISSENSE mutations of HRR or other DDR pathways were defined as mutations in this study. Mutations were performed only in the therapeutic-, prognostic-, or diagnostic-related regions according to standards and guidelines for the interpretation and reporting of sequence variants in cancer by the American College of Medical Genetics and Genomics (ACMG).

### Statistical analysis

Overall survival (OS) was plotted to obtain and compare survival through the Kaplan–Meier curve (log-rank test). Univariate and multivariate Cox proportional hazard regression analyses were used to quantify the hazard ratio of various characteristics. The differences between the two groups used by Student’s t-test and Mann–Whitney U test for normally distributed continuous variables were determined. The correlation of two categorical variables through Fisher’s exact test or the chi-square test was identified. According to all reports, P values were two-tailed and P < 0.05 was considered significant unless otherwise specified. All analyses and graphs in this study were processed through R 3.6.0.

## Results

### HRR pathway gene mutations may be a biomarker to predict the efficacy of anti-CTLA-4 therapy in melanoma

**Figure 2:**
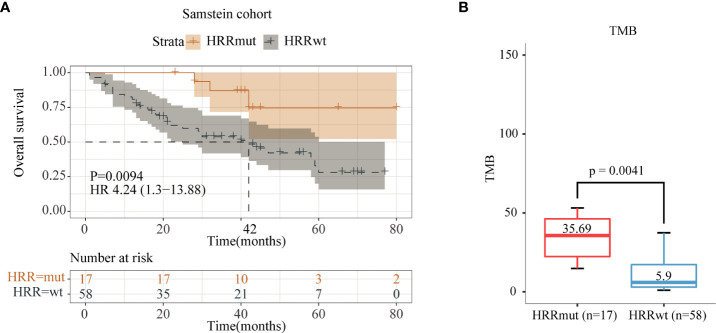
Analysis of immune indices and prognosis of the Samstein2018 cohort in anti-CTLA-4 therapy. **(A)** Kaplan–Meier survival curves of OS in the immunotherapy-treated patients with or without HRR mutations. **(B)** TMB levels between the HRR-mutant and HRR wild-type groups.

Data analysis was performed using the Samstein2018 cohort with 75 advanced melanoma patients who received anti-CTLA-4 therapy, and the results showed that 17 patients (22.7%) harbored HRR mutations (HRRmut). Tumor mutation burden (TMB) levels in HRRmut patients were significantly higher than those in HRR wild-type (HRRwt) patients (35.69 vs. 5.90 Muts/Mb, P = 0.0041, **Figure 2B**). Patients receiving anti-CTLA4 therapy with HRRmut had significantly better median overall survival (mOS) than those with the HRRwt (NR versus 42 months, hazard ratio (HR) =4.24, 95% CI 1.30–13.88, P = 0.0094, **Figure 2A**).

**Figure 3:**
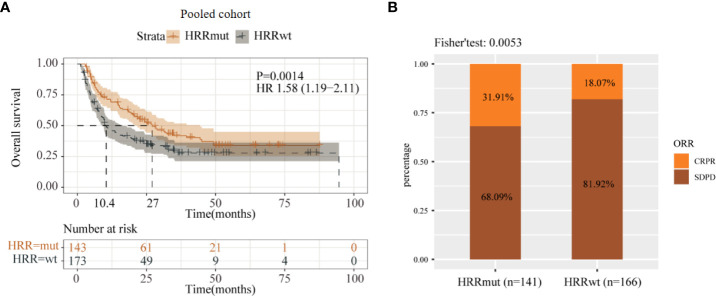
Analysis of prognosis of the pooled cohort in anti-CTLA-4 therapy. **(A)** Kaplan–Meier survival curves of OS in immunotherapy-treated patients with or without HRR mutations. **(B)** Efficacy of immunotherapy between the HRR mutant and HRR wild-type groups. Some patients were not included in efficacy analysis due to lack of response information.

In order to validate the predictive value of HRR pathway mutation for the efficacy of anti-CTLA4 therapy in melanoma, it was also performed using a pooled cohort receiving anti-CTLA-4 therapy for the data analysis. According to the results, 143 (45.3%) patients of the pooled cohort harbored HRRmut. Similar to the Samstein2018 cohort, the HRRmut group was significantly correlated with longer mOS (27.0 versus 10.4 months, HR = 1.58, 95% CI 1.19–2.11, P = 0.0014) (**Figure 3A**). Moreover, the objective response rate (ORR) of anti-CTLA-4 therapy was 31.9% for the patients with HRRmut and 18.1% for the HRRwt group (P = 0.0053) (**Figure 3B**).

### HRR pathway gene mutations were not associated with the efficacy of anti-PD-1/L1 therapy in melanoma

**Figure 4:**
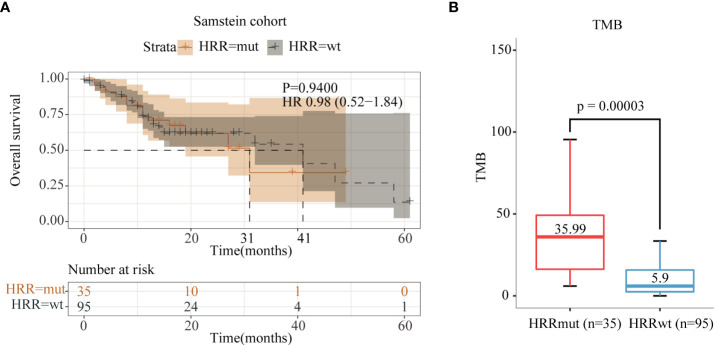
Analysis of immune indices and prognosis of the Samstein2018 cohort in anti-PD-1/L1 therapy. **(A)** Kaplan–Meier survival curves of OS in the immunotherapy-treated patients with or without HRR mutations. **(B)** TMB levels between the HRR-mutant and HRR wild-type groups.

Anti-PD-1 therapy can lengthen both PFS and OS in advanced melanoma patients as a first-line drug recommended by the NCCN guidelines, whose clinical performance is superior to anti-CTLA-4 therapy (29). In order to explore whether HRRmut is also associated with the benefit of anti-PD-1/L1 therapy, we performed data analysis on the Samstein2018 cohort with 130 advanced melanoma patients who received anti-PD-1/L1 therapy, of which 35 patients (26.9%) harbored HRRmut. Moreover, HRRmut (35.99 Muts/Mb) was associated with higher tumor mutation burden (TMB) (P < 0.0001) than HRRwt (5.90 Muts/Mb) (**Figure 4B**), similar with the patients with anti-CTLA-4 therapy. However, in terms of prognosis in anti-PD-1/L1 therapy, the HRRmut patients had no significantly improved mOS than the HRRwt group (41 versus 31 months, HR = 0.98, 95% CI 0.52–1.84, P = 0.94) (**Figure 4A**).

**Figure 5:**
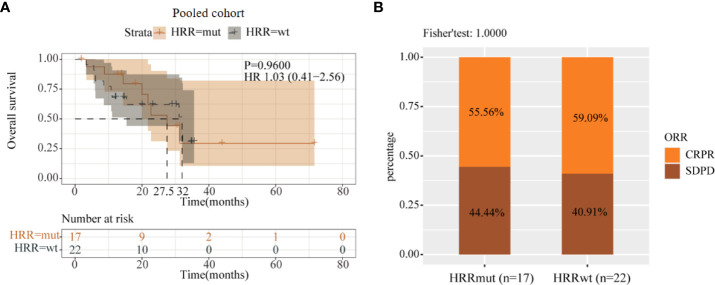
Analysis of prognosis of the pooled cohort in anti-PD-1/L1 therapy. **(A)** Kaplan–Meier survival curves of OS in immunotherapy-treated patients with or without HRR mutations. **(B)** Efficacy of immunotherapy between the HRR mutant and HRR wild-type groups. Some patients were not included in efficacy analysis due to lack of response information.

To further validate the results, we performed data analysis using a pooled cohort of 39 melanoma patients who received anti-PD-1/L1 therapy. As a result, 17 patients (43.6%) harbored HRRmut in this pooled cohort, and there was also no significant difference in mOS between the HRRmut and HRRwt groups (20.75 vs. 32.0 months; HR = 1.03; 95% CI, 0.41–2.56; P = 0.96; **Figure 5A**). Moreover, the ORR also had no significant difference between HRRmut and HRRwt groups (P = 1.0) (**Figure 5B**). This suggests that there is no correlation between HRR pathway genes and the clinical benefits of anti-PD-1/L1 therapy in advanced melanoma, which might have a specific relationship with anti-CTLA-4 therapy.

### The association of HRR pathway gene mutation and different types of ICI therapy

**Figure 6:**
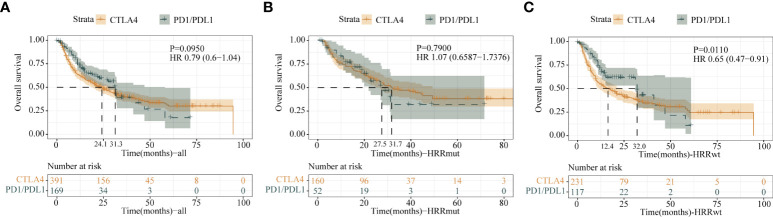
Kaplan–Meier survival curves of OS of the **(A)** all-patient group, **(B)** HRR mutation group, and **(C)** HRR wild-type group between anti-CTLA-4 therapy and anti-PD1/L1 therapy.

In order to better assist in the selection of therapy, we compared the correlation between two different treatment modalities and HRRmut. Comparing the efficacy of two therapies in the total cohort formed by all the cohorts, although there was no significant difference in the mOS between the CTLA-4 and PD-1/L1 inhibitors in all patients (HR = 0.79; 95% CI, 0.60–1.04; P = 0.095) and the HRRmut group (HR = 1.07; 95% CI, 0.66–1.74; P = 0.79), the number of mOS in the HRRmut group with CTLA-4 tended to be better than in the PD-1/L1 group (mOS: 31.7 vs. 27.5 months) (**Figures 6A, B**). However, the OS in the HRRwt group with CTLA-4 was significantly worse than in the PD-1/L1 group (mOS: 12.4 vs. 32.0 months, HR = 0.65; 95% CI, 0.47–0.91; P = 0.01) (**Figure 6C**). This suggests that anti-CTLA-4 therapy may also be a first-line treatment option in patients with HRRmut as well as anti-PD-1/L1 therapy.

### The association of HRR pathway gene mutation and immune microenvironment

To assess the impact of HRRmut on the transcription of immune-related genes, we integrated gene expression data of 471 patients from TCGA-SKCM cohort. TIMER tools were employed to analyze the gene expression data to evaluate immune cells of tumor immune microenvironment infiltration in immunotherapy-treated patients. Among 44 genes in the HRR pathway analyzed in this study, 29 (65.9%) were associated with the reshaping of the immunological microenvironment, in which 22 (50.0%) gene mutations were positively associated with better immune cell infiltration and six (13.6%) were negatively associated with it. Moreover, mutations of ERCC1, POLD1, RFC2, and TP53BP1 were significantly correlated with five or more types of immune cells, in which POLD1 and POLD3 have a positive or negative effect on different immune cells (**Supplementary Table 3**).

In addition, we also analyzed which gene mutations were most strongly associated with each type of immune cell. The infiltration of CD8^+^ T cells had the most significant difference in ERCC1 mutation and wild type (P = 0.0035). Moreover, the infiltration of CD4^+^ T cells was in RBBP8 (P = 0.0048), Tregs in POLD1 (P = 0.001), B cells in MUS81 (P = 0.0096), macrophages in BRCA1 (P = 0.003), NK cells in RFC2 (P = 0.0047), and DC cells in XRCC3 (P = 0.031) which had the most significant difference between mutation and wild type (Supplementary **Figure 1**, **Supplementary Table 3**).

### Co-mutations of HRR with other DDR pathway genes may not predict the efficacy of anti-CTLA-4 therapy in melanoma

The DDR pathway includes seven different pathways (including HRR). To explore more possibilities for immunotherapy-related biomarkers in advanced melanoma, we also performed data analysis on the predictive efficacy of HRR combined with other pathways. The results showed that mutations of HRR combination with any other gene of the DDR pathway cannot predict the efficacy for anti-CTLA-4 therapy.

### The prognostic role of HRR alterations

We evaluated whether HRR status was a prognostic factor using the survival data and sequencing data of 287 previously untreated advanced melanoma patients in TCGA database. The results showed that HRR alteration status did not appear to correlate with the OS (HR = 1.00; 95% CI, 0.70–1.43, P = 0.98; **Supplementary Figure 2**). These results suggested that HRR alteration status was not a prognostic factor for advanced melanoma.

## Discussion

In this study, we analyzed several public cohorts and found that HRR mutation might be a potential predictor of clinical benefit to anti-CTLA-4 therapy in advanced melanoma. Although there have been some studies about THE HRR gene in melanoma, to the best of our knowledge, this is the first systematic study to examine the association between HRR and immunotherapy to date.

As early as 2018, Min et al. reported the correlation between DDR gene and immunotherapy in advanced urethral carcinoma, followed by similar reports in other cancers (13, 24). The mechanism is that altered DNA damage responses mediated by exposure to cytotoxic agents or loss of normal DNA repair ability may contribute to antitumor immunity mediated by the STING pathway (30). When cGAMP synthase (cGAS) interacts with cell-soluble DNA and catalyzes the synthesis of cGAMP, the STING pathway is activated. CGAMP is a circulating dinucleotide. After being activated as a second messenger, STING undergoes conformational change, which causes it to cross from the endoplasmic retina to the peritoneal endocrine body, where it is activated and undergoes phosphorescence by TBK1. TBK1 also phosphates interferon regulatory factor 3 (IRF3), which is transferred to the nucleus to drive transcription of type I interferon (IFN) genes. Finally, the activation of STING pathways in antigen-presenting cells (APCs) in the tumor microenvironment drives T cells to stimulate tumor-associated antigens and promote the occurrence of antitumor immunity (31–33). Although the mechanism is not fully elucidated, *in vivo* studies also demonstrated that this process is required for innate immune sensing and T-cell initiation of antitumor activity, and CGAS-STING is also required for the antitumor effects of ICI (14). In 2021, Kim et al. explored the correlation between DDR pathway genes and immunity in melanoma and found that DDR pathway genes may be a potential biomarker for predicting the efficacy of immunotherapy in melanoma, but the study did not discuss the different DDR pathway genes (34). The results of this study not only are consistent with the results of previous studies but also provide evidence for the prediction of HRR pathway gene for immunotherapy of melanoma.

In the immune microenvironment, previous studies have shown that the DDR pathway can activate and upregulate most immune cells including T cells, B cells, macrophages, and Tregs through the activation of CGAS-STING, making these patients more likely to benefit from immunity (35, 36). This is consistent with the results of immunity analysis of most genes in the HRR pathway in this study. However, there are still some genes in the pathway that only have a negative regulation on immune cells, and POLD family genes have different positive and negative regulations on different immune cells. In fact, the immune function of each gene in the HRR pathway is also different, and a study of hepatocellular carcinoma showed that further classification of patient subtype by genomic characteristics can better distinguish and explain its influence on immune cell infiltration (37, 38). In conclusion, the HRR gene in the DDR pathway is not completely similar to the immune characteristics of the DDR pathway, which is worth more research on it.

Previous studies have shown that deficiencies in a variety of DDR pathways including HRR can result in increased TMB (24, 39). In 2021, a study of immune-related biomarkers in pan-cancer patients showed that HRR pathway mutations were associated with high TMB levels in multiple tumor species, and the combination of TMB and HR-DDR may be a potential biomarker for predicting ICIs’ efficacy (40). A retrospective review of the Checkmate 066 and Checkmate 067 studies found that melanoma patients with higher TMB had better immunotherapy efficacy in both studies (41), which may be one of the reasons for better immunotherapy efficacy in patients with HRR in this study.

This study also explored the correlation between different ICI treatment drugs and HRR pathway gene mutations. Results showed that melanoma patients with HRRmut had better curative effect in anti-CTLA-4 therapy than HRRwt, but there was no significant difference in anti-PD-1/PD-L1 therapy. As discussed above, the immune mechanisms affecting patients with HRRmut are quite complex and unclear. In addition to TMB, the infiltrating state of different immune cells, the quality of antigen, and HLA-DR expression all affect the efficacy of CTLA-4 inhibitors (42, 43). Therefore, TMB is not the only predictor of immunotherapy efficacy. In this study, for patients with HRRmut (P = 0.79) there was no significant difference in mOS (**Figure 6B**), whether using the CTLA-4 inhibitor or the PD-1/PD-L1 inhibitor. It is well known that in NCCN guideline recommendations, PD-1/PD-L1 inhibitors are first-line recommendations, while CTLA-4 inhibitors are not (29, 43), which is similar to the result of OS in the HRRwt and all-patient groups. In contrast, the mOS value of CTLA-4 inhibitors was better than that of PD-1/L1 inhibitors in the HRRmut group (31.7 vs. 27.5 months). With the above results, anti-CTLA-4 therapy may also be a first-line option for patients with HRR mutations.

In order to find more predictive opportunities, we also explored the combination of HRR and other DDR pathway genes to predict the efficacy of anti-CTLA-4 therapy. A previous study showed that the co-mutations of HRR/MMR and HRR/BER can predict the immunotherapy efficacy in non-small cell lung cancer (44). However, in our study, there was none who had a predictive efficacy in the seven pathways of DDR. In addition, in our study HRR mutation rates were not similar between the pooled cohort and the Samstein cohort, which may be related to the patient population. In fact, previous studies have shown that the HRR mutation rate in Chinese melanoma is 40%, similar to that in the pooled cohort, and the mutation rate of TCGA database is 26%, similar to the Samstein cohort (45). However, due to the limitations of patient information disclosure in the public cohort we included, further analysis was not possible.

There are some limitations in our study. The ICI-treated cohorts incorporated into the study were from multiple study centers; thus, the pooled analysis might introduce biases caused by the difference in ICI regimen, dose usage, treatment cycle, etc., and the absence of specific limitations on the number of treatment lines and duration of treatment for patients in these cohorts resulted in differences in survival time among cohorts. Moreover, the difference in the number of patients between the two therapies may affect the results of efficacy comparison. As for the mechanism of HRR as an independent gene on immunity, we can only preliminarily explore its correlation with the immune microenvironment through bioinformatics analysis; the level of evidence is inadequate. Therefore, prospective studies are needed to confirm our observations. In addition, the mechanism underlying the clinical benefit of HRRmut melanoma to anti-CTLA-4 needs to be further interpreted by basic research.

HRR pathway gene mutation was associated with a higher TMB level and immunotherapeutic effect. HRR may serve as an independent predictor of anti-CTLA-4 therapy efficacy in patients with advanced melanoma, and their clinical value deserves further investigation.

## Data availability statement

Publicly available datasets were analyzed in this study. This data can be found here: Availability of data and materials TCGA data used in the article are available from https://www.cbioportal.org/ with cancer types under PanCancer Altas subtype. Samstein data are available from https://www.cbioportal.org/study/summary?id=tmb_mskcc_2018; Allen data are available from https://www.cbioportal.org/study/summary?id=skcm_dfci_2015; Miao data are available from https://www.cbioportal.org/study/summary?id=mixed_allen_2018; Hugo data are available from https://www.cbioportal.org/study/summary?id=mel_ucla_2016; Snyder data are available from https://www.cbioportal.org/study/summary?id=skcm_mskcc_2014. The datasets supporting the conclusions and other relevant data of this article are available from the corresponding authors upon reasonable request. All custom code used in this work is available from the corresponding authors upon reasonable request.</b>.

## Ethics statement

This research was approved by the Research Ethics Committee of the Nanfang Hospital, Southern Medical University and individual consent for this retrospective analysis was waived.

## Author contributions

Conception and design: ZY, CZ, CY. Acquisition of data: ML, XH, YP, JG. Analysis and interpretation of data: ZY, XH, ML, XH. Writing, review, and/or revision of the manuscript: XH, YZ. Study supervision: CY, MH. Final approval of manuscript: All authors.

## Funding

This study was supported by the Open Project of State Key Laboratory of Respiratory Diseases (SKLRD-OP-202111 to CY) and the Undergraduate Science and Technology Innovation Project of Guangzhou Medical University (2021A008 to ZY).

## Conflict of interest

Author Xue Hu, Yating Zheng, and Mengli Huang are employed by the company 3D Medicines Inc.

The remaining authors declare that the research was conducted in the absence of any commercial or financial relationships that could be construed as a potential conflict of interest.

## Publisher’s note

All claims expressed in this article are solely those of the authors and do not necessarily represent those of their affiliated organizations, or those of the publisher, the editors and the reviewers. Any product that may be evaluated in this article, or claim that may be made by its manufacturer, is not guaranteed or endorsed by the publisher.

## Abbreviations

ICIs, Immune checkpoint inhibitors; DDR, DNA damage response; HRR, Homologous recombination repair; MMR, Mismatch repair; BER, Base excision repair; FA, Fanconi anemia; NER, Nucleotide excision repair; NEJ, Non-homologous end-joining; TLS, DNA translesion synthesis; SNVs, Single nucleotide variants; HRRwt, HRR pathway gene wild-type; HRRmut, Somatic HRR pathway gene mutation; PARP, Polyadenosine diphosphate-ribose polymerase; PD-L1, Programmed Cell Death-Ligand 1; PD-1, Programmed Cell Death 1; CTLA-4, Cytotoxic T lymphocyte-associated antigen-4; TMB, Tumor mutation burden; ORR, Objective response rate; OS, Overall survival; STING, Stimulator of interferon genes.

## References

[1] Robert C Lanoy E Besse B. One or two immune checkpoint inhibitors? Cancer Cell (2019) 36:579–81. doi: 10.1016/j.ccell.2019.11.005 31951559

[2] Sosman JA Haanen JB Gonzalez R Robert C Ph D Schadendorf D. Improved survival with ipilimumab in patients with metastatic melanoma. N Engl J Med (2010), 363(8):711–23. doi: 10.1056/NEJMoa1003466 PMC3549297 20525992

[3] Schachter J Ribas A Long GV Arance A Grob JJ Mortier L. Pembrolizumab versus ipilimumab for advanced melanoma: final overall survival results of a multicentre, randomised, open-label phase 3 study (KEYNOTE-006). Lancet (2017) 390:1853–62. doi: 10.1016/S0140-6736(17)31601-X 28822576

[4] Wolchok JD Chiarion-Sileni V Gonzalez R Rutkowski P Grob J-J Cowey CL. Overall survival with combined nivolumab and ipilimumab in advanced melanoma. N Engl J Med (2017) 377:1345–56. doi: 10.1056/nejmoa1709684 PMC5706778 28889792

[5] Herrscher H Robert C. Immune checkpoint inhibitors in melanoma in the metastatic, neoadjuvant, and adjuvant setting. Curr Opin Oncol (2020) 32:106–13. doi: 10.1097/CCO.0000000000000610 31876547

[6] Imbert C Montfort A Fraisse M Marcheteau E Gilhodes J Martin E. Resistance of melanoma to immune checkpoint inhibitors is overcome by targeting the sphingosine kinase-1. Nat Commun (2020) 11:1–14. doi: 10.1038/s41467-019-14218-7 31974367 PMC6978345

[7] Snyder A Makarov V Merghoub T Yuan J Zaretsky JM Desrichard A. Genetic basis for clinical response to CTLA-4 blockade in melanoma. N Engl J Med (2014) 371:2189–99. doi: 10.1056/nejmoa1406498 PMC4315319 25409260

[8] Van Allen EM Miao D Schilling B Shukla SA Blank C Zimmer L. Genomic correlates of response to CTLA-4 blockade in metastatic melanoma. Science (2016) 350:207–11. doi: 10.1126/science.aad0095.Genomic PMC5054517 26359337

[9] Williams B Jalilianhasanpour R Matin N Fricchione GL Sepulcre J Keshavan MS. Individual differences in corticolimbic structural profiles linked to insecure attachment and coping styles in motor functional ne 230-237. (2018) 362(6411):eaar3593. doi: 10.1126/science.aar3593.Pan-tumor PMC6005758 29702433

[10] Lord CJ Ashworth A. The DNA damage response and cancer therapy. Nature (2012) 481:287–94. doi: 10.1038/nature10760 22258607

[11] Vidula N Horick NK Blouch E Rivera A Basile E Fax R. Phase II trial of a PARP inhibitor in somatic BRCA mutant metastatic breast cancer. J Clin Oncol (2020) 38:TPS1113–TPS1113. doi: 10.1200/jco.2020.38.15_suppl.tps1113

[12] Dorling L Carvalho S Allen J González-Neira A Luccarini C Wahlström C. Breast cancer risk genes — association analysis in more than 113,000 women. N Engl J Med (2021) 384:428–39. doi: 10.1056/nejmoa1913948 PMC7611105 33471991

[13] Teo MY Seier K Ostrovnaya I Regazzi AM Kania BE Moran MM. Alterations in DNA damage response and repair genes as potential marker of clinical benefit from PD-1/PD-L1 blockade in advanced urothelial cancers. J Clin Oncol (2018) 36:1685–94. doi: 10.1200/JCO.2017.75.7740 PMC6366295 29489427

[14] Chabanon RM Rouanne M Lord CJ Soria JC Pasero P Postel-Vinay S. Targeting the DNA damage response in immuno-oncology: developments and opportunities. Nat Rev Cancer (2021) 21:701–17. doi: 10.1038/s41568-021-00386-6 34376827

[15] Scarbrough PM Weber RP Iversen ES Brhane Y Amos CI Kraft P. A cross-cancer genetic association analysis of the DNA repair and DNA damage signaling pathways for lung, ovary, prostate, breast, and colorectal cancer. Cancer Epidemiol Biomarkers Prev (2016) 25:193–200. doi: 10.1158/1055-9965.EPI-15-0649 26637267 PMC4713268

[16] Hussain M Mateo J Fizazi K Saad F Shore N Sandhu S. Survival with olaparib in metastatic castration-resistant prostate cancer. N Engl J Med (2020) 383:2345–57. doi: 10.1056/nejmoa2022485 32955174

[17] Karzai F VanderWeele D Madan RA Owens H Cordes LM Hankin A. Activity of durvalumab plus olaparib in metastatic castration-resistant prostate cancer in men with and without DNA damage repair mutations. J Immunother Cancer (2018) 6:1–12. doi: 10.1186/s40425-018-0463-2 30514390 PMC6280368

[18] Stewart MD Vega DM Arend RC Baden JF Barbash O Beaubier N. Homologous recombination Deficiency : Concepts , definitions , and assays. Oncologist (2022), 27(3):167–74. doi: 10.1093/oncolo/oyab053 35274707 PMC8914493

[19] Aoude LG Xu M Zhao ZZ Kovacs M Palmer JM Johansson P. Assessment of PALB2 as a candidate melanoma susceptibility gene. PloS One (2014) 9(6):e100683. doi: 10.1371/journal.pone.0100683 24949998 PMC4065098

[20] Fan Y Ying H Wu X Chen H Hu Y Zhang H. The mutational pattern of homologous recombination (HR)-associated genes and its relevance to the immunotherapeutic response in gastric cancer. Cancer Biol Med (2020) 17:1002–13. doi: 10.20892/j.issn.2095-3941.2020.0089 PMC7721103 33299649

[21] Zhou P Wu X Chen H Hu Y Zhang H Wu L. The mutational pattern of homologous recombination-related (HRR) genes in Chinese colon cancer and its relevance to immunotherapy responses. Aging (Albany NY) (2021) 13:2365–78. doi: 10.18632/aging.202267 PMC7880324 33318301

[22] Pellegrino B Musolino A Llop-Guevara A Serra V De Silva P Hlavata Z. Homologous recombination repair deficiency and the immune response in breast cancer: A literature review. Transl Oncol (2020) 13:410–22. doi: 10.1016/j.tranon.2019.10.010 PMC6948367 31901781

[23] Creeden JF Nanavaty NS Einloth KR Gillman CE Stanbery L Hamouda DM. Homologous recombination proficiency in ovarian and breast cancer patients. BMC Cancer (2021) 21:1–12. doi: 10.1186/s12885-021-08863-9 34711195 PMC8555001

[24] Ricciuti B Recondo G Spurr LF Li YY Lamberti G Venkatraman D. Impact of DNA damage response and repair (DDR) gene mutations on efficacy of PD-(L)1 immune checkpoint inhibition in non–small cell lung cancer. Clin Cancer Res (2020) 26:4135–42. doi: 10.1158/1078-0432.CCR-19-3529 32332016

[25] Seeber A Zimmer K Kocher F Puccini A Xiu J Nabhan C. Molecular characteristics of BRCA1/2 and PALB2 mutations in pancreatic ductal adenocarcinoma. ESMO Open (2020) 5:1–7. doi: 10.1136/esmoopen-2020-000942 PMC7684832 33229504

[26] Miao D Margolis CA Gao W Voss MH Li W Dylan J. Genomic correlates of response to immune checkpoint therapies in clear cell renal cell carcinoma Diana. Science (2018) 359:801–6. doi: 10.1126/science.aan5951.Genomic PMC6035749 29301960

[27] Samstein RM Lee CH Shoushtari AN Hellmann MD Shen R Janjigian YY. Tumor mutational load predicts survival after immunotherapy across multiple cancer types. Nat Genet (2019) 51:202–6. doi: 10.1038/s41588-018-0312-8.Corresponding PMC6365097 30643254

[28] Hugo W Zaretsky JM Sun L Song C Homet B Hu-lieskovan S. Genomic and transriptomic features of anti-PD1 response. Cell (2017) 165:35–44. doi: 10.1016/j.cell.2016.02.065.Genomic PMC4808437 26997480

[29] NCCN. The NCCN melanoma: cutaneous clinical practice guidelines in oncology (version 2.2022)[EB/OL] Fort Washington: NCCN,2022[2022-01-26]. Available at: http://www.nccn.org.

[30] Mouw KW Goldberg MS Konstantinopoulos PA D'Andrea AD. DNA Damage and repair biomarkers of immunotherapy response. Cancer Discov (2017) 176:139–48. doi: 10.1158/2159-8290.CD-17-0226.DNA PMC5659200 28630051

[31] Glickman LH Corrales L Kanne DB Kasibhatla S Li J. Abstract SY39-02: Direct activation of STING in the tumor microenvironment leads to potent and systemic tumor regression and immunity. Cell Rep (2016) 11:SY39–02-SY39-02. doi: 10.1158/1538-7445.am2016-sy39-02 PMC4440852 25959818

[32] Chen Q Sun L Chen ZJ. Regulation and function of the cGAS-STING pathway of cytosolic DNA sensing. Nat Immunol (2016) 17:1142–9. doi: 10.1038/ni.3558 27648547

[33] Klarquist J Hennies CM Lehn MA Reboulet RA Feau S. STING-mediated DNA sensing promotes antitumor and autoimmune responses to dying cells. J Immunol (2014) 23:1–7. doi: 10.4049/jimmunol.1401869.STING-mediated PMC4258444 25385820

[34] Kim KB Soroceanu L de Semir D Millis SZ Ross J Vosoughi E. Prevalence of homologous recombination pathway gene mutations in melanoma: Rationale for a new targeted therapeutic approach. J Invest Dermatol (2021) 141:2028–36.e2. doi: 10.1016/j.jid.2021.01.024 33610559

[35] Nastasi C Mannarino L D’incalci M. DNA Damage response and immune defense. Int J Mol Sci (2020) 21:1–28. doi: 10.3390/ijms21207504 PMC7588887 33053746

[36] Zhu Y An X Zhang X Qiao Y Zheng T Li X. STING: A master regulator in the cancer-immunity cycle. Mol Cancer (2019) 18:1–15. doi: 10.1186/s12943-019-1087-y 31679519 PMC6827255

[37] Nandi B Talluri S Kumar S Yenumula C Gold JS Prabhala R. The roles of homologous recombination and the immune system in the genomic evolution of cancer. J Transl Sci (2019) 5(2):10.15761/JTS.1000282. doi: 10.15761/JTS.1000282.The PMC6411307 30873294

[38] Lin H Xie Y Kong Y Yang L Li M. Identification of molecular subtypes and prognostic signature for hepatocellular carcinoma based on genes associated with homologous recombination deficiency. Sci Rep (2021) 11:1–15. doi: 10.1038/s41598-021-03432-3 34912005 PMC8674316

[39] Xiao Y Lu D Lei M Xie W Chen Y Zheng Y. Comprehensive analysis of DNA damage repair deficiency in 10,284 pan-cancer study. Ann Transl Med (2021) 9:1661–1. doi: 10.21037/atm-21-5449 PMC8667116 34988170

[40] Wang HY Deng L Li YQ Zhang X Long YK Zhang X. Pan-cancer analysis of tumor mutational burden and homologous recombination DNA damage repair using targeted next-generation sequencing. Cancer Res Treat (2021) 53:973–82. doi: 10.4143/crt.2020.798 PMC8524032 33677848

[41] Hodi FS Wolchok JD Schadendorf D Larkin J Long GV Qian X. TMB and inflammatory gene expression associated with clinical outcomes following immunotherapy in advanced melanoma. Cancer Immunol Res (2021) 9:1202–13. doi: 10.1158/2326-6066.CIR-20-0983 PMC9414280 34389558

[42] Wolchok JD Saenger Y. The mechanism of anti-CTLA-4 activity and the negative regulation of T-cell activation. Oncologist (2008) 13:2–9. doi: 10.1634/theoncologist.13-s4-2 19001145

[43] Hosseini A Gharibi T Marofi F Babaloo Z Baradaran B. CTLA-4: From mechanism to autoimmune therapy. Int Immunopharmacol (2020) 80:106221. doi: 10.1016/j.intimp.2020.106221 32007707

[44] Wang Z Zhao J Wang G Zhang F Zhang Z Zhang F. Comutations in DNA damage response pathways serve as potential biomarkers for immune checkpoint blockade. Cancer Res (2018) 78:6486–96. doi: 10.1158/0008-5472.CAN-18-1814 30171052

[45] Liu H Zhang Y Ding F Zhang Y Liang X Lou F. Frequency of homologous recombination deficiency gene mutations in melanoma and its relevance to the immunotherapeutic response. J Clin Oncol (2021) 39:e15073–3. doi: 10.1200/jco.2021.39.15_suppl.e15073

