# Supplementary material for: Homologous recombination repair gene mutations as a predictive biomarker for immunotherapy in patients with advanced melanoma
Source: Front Immunol. 2022 Aug 3;13:871756. doi: 10.3389/fimmu.2022.871756 (PMC9381822; doi:10.3389/fimmu.2022.871756)
Supplement: Supplementary file 1 [file DataSheet_1.pdf]

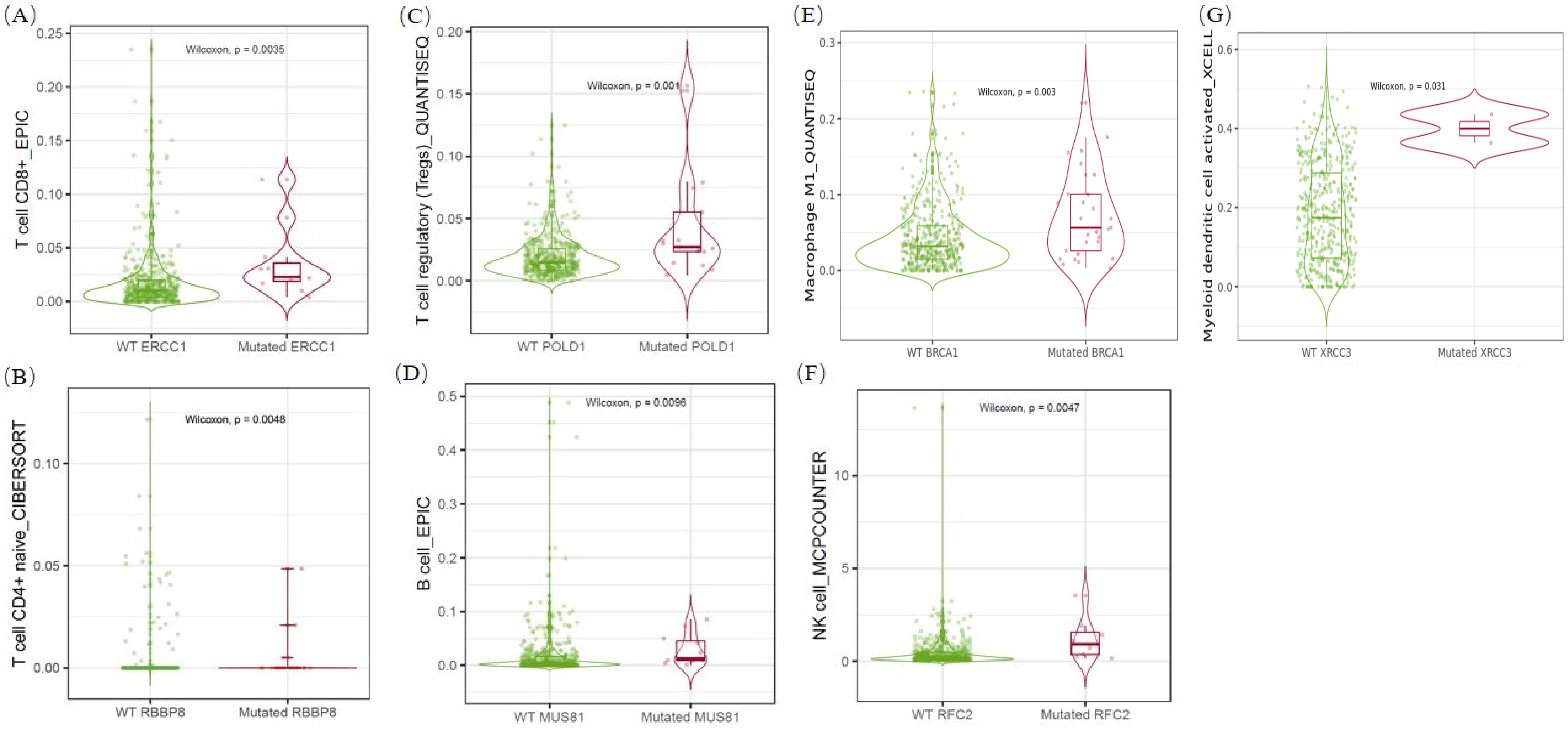

Supplementary Figure 1: Comparison of different degree of immune cell infiltration between HRR mutation and HRR wild-type group, the most significant difference included (A) CD8+T cell in ERCC1, (B) CD4+T cell in RBBP8, (C) Tregsin POLD1, (D) B cell in MUS81, (E) M1 macrophage in BRCA1, (F) NK cell in RFC2, and (G) DC cell in XPCC3..
