# Supplementary material for: Homologous recombination repair gene mutations as a predictive biomarker for immunotherapy in patients with advanced melanoma
Source: Front Immunol. 2022 Aug 3;13:871756. doi: 10.3389/fimmu.2022.871756 (PMC9381822; doi:10.3389/fimmu.2022.871756)
Supplement: Supplementary file 2 [file DataSheet_2.pdf]

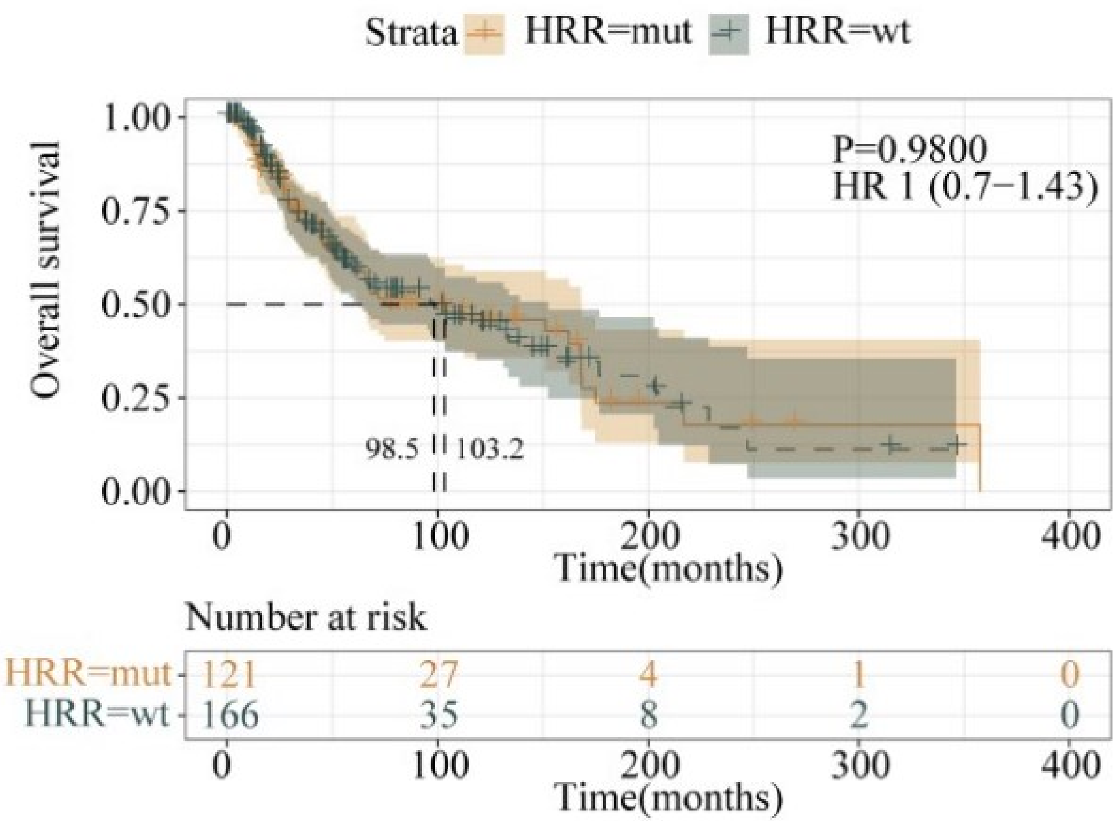

Supplementary Figure 2: Analysis of prognosis of the TCGA. Kaplan-Meier survival curves of OS with or without HRR mutations.
